# Case report and literature review: Primary leiomyosarcoma of the bone in the trochanteric region of the femur

**DOI:** 10.3389/fsurg.2022.1045307

**Published:** 2023-01-10

**Authors:** Zhonghan Wu, Li Cheng, Qiliang Cao, Shuming Ye, Shuisheng Yu, Min Sun, Juehua Jing

**Affiliations:** ^1^ Department of Orthopaedics, The Second Hospital of Anhui Medical University, Hefei, China; ^2^ Institute of Orthopaedics, Research Center for Translational Medicine, The Second Hospital of Anhui Medical University, Hefei, China

**Keywords:** leiomyosarcoma, bone neoplasms, femur, invasive, surgical treatment, joint replacement, case report

## Abstract

**Background:**

Primary leiomyosarcoma of the bone (LMSB) is an extremely rare, invasive, and highly destructive primary osteosarcoma with limited treatment options and poor prognosis. Only a few case reports of LMSB have been described because of its rarity. Therefore, clinicians have a limited understanding of its diagnosis, treatment, and prognosis, and the final diagnosis depends on histopathological findings. In this report, we describe a rare case of primary LMSB in the trochanteric region of the femur. Reporting this case may increase the dissemination and understanding of information regarding LMSB and provide a reference for the diagnosis and treatment of similar cases.

**Case presentation:**

A 63-year-old woman presented with pain and limited movement of the left hip, which had lasted for 3 months, with no history of trauma or illness. Plain radiography and computed tomography revealed a solitary osteolytic lesion in the trochanteric area of the left femur with focal cortical destruction. Magnetic resonance imaging findings suggested invasion of the lesion into the bone cortex, forming a soft tissue mass, although no distant positive findings were observed on a whole-body bone scan. A bone tumor puncture biopsy was performed to obtain a final diagnosis, and histopathological evaluation revealed left femoral intertrochanteric leiomyosarcoma, classified as G1T2M0 and staged as IB (extracompartmental low-grade malignant) according to the Enneking staging system. Thus, we performed extensive debridement and left hip arthroplasty. Postoperative chemotherapy was administered, and the patient was followed up for 4 years. Four years later, the patient's left hip pain had resolved, joint activity was good, and no signs of recurrence or distant metastasis of the bone tumor were noted.

**Conclusion:**

For proximal femoral Enneking stage IB LMSB, extensive tumor resection combined with tumor prosthesis replacement may be an effective treatment method to prolong the patient's lifespan and to restore joint function.

## Introduction

Primary leiomyosarcoma of the bone (LMSB) is a rare bone tumor first reported by Evans and Sanerkin in 1965 and accounts for approximately 0.06% of all primary bone tumors (1). Primary LMSB lesions comprise <0.7% of all primary bone malignancies (2). They are thought to originate from small vascular smooth muscle cells in the bone cortex or pluripotent mesenchymal stem cells in the bone. They are pathologically characterized as osteosarcomas with smooth muscle-like differentiation (3). Leiomyosarcoma commonly occurs in the retroperitoneum, subcutaneous tissue of the extremities, intraabdominal space, and gastrointestinal, uterine, and other deep soft tissues (4). LMSB usually occurs in the long tubular bones of the lower extremities (tibia and femur), and approximately 70% of cases occur in the knee joint (distal femur and proximal tibia) (5). Its occurrences in the hip joint, spine, and trochanteric area of the femur have been rarely reported, especially in the last 10 years (Supplementary Table S1).

The early diagnosis and treatment of LMSB are difficult (6). LMSB mostly affects middle-aged and older patients and is usually characterized by pain and swelling of the affected limbs (7). Moreover, pathological fractures may occur in 30% of cases (7). The findings on magnetic resonance imaging (MRI) are easily confused with those of osteolytic osteosarcoma and metastatic leiomyosarcoma, and diagnosing LMSB depends on pathological methods (7). The tumor has high metastatic and recurrence rates (8, 9). From the perspective of Enneking surgical staging, high tumor grade, tumor diameters exceeding 5 cm, and early metastasis increase the long-term mortality of LMSB (10). Therefore, early diagnosis can improve the survival prognosis. As a malignant bone tumor, the most basic treatment method for LMSB is surgical resection, which can be classified into two types: limb salvage and amputation, both of which aim to completely remove the pathological tissue (11). For malignant bone tumors near joints, patients often undergo tumor prosthesis replacement surgery (12, 13). Tumor prosthesis replacement meets the marginal requirements of an extensive resection of tumor tissue and retains joint function (11). These tumors are relatively resistant to radiotherapy and chemotherapy, and their prognosis is complicated (14).

There have only been a few detailed reports of LMSB in the hip joint. Herein, we present a rare case of an older female patient with primary leiomyosarcoma in the trochanter of the left femur with a good prognosis.

## Case description

### Chief complaints

A 63-year-old woman who complained of pain and limited left hip movement for the previous 3 months was hospitalized in November 2017. In the early stages of the disease (August 2017), the patient experienced non-specific, intractable hip pain and limited movement without external trauma or injury. The pain was aggravated by exercise and was more severe at night, accompanied by claudication and difficulty turning over in bed. Radiography performed at a local county hospital showed a bone tumor in the left proximal femur; hence, the patient was referred to our hospital for treatment.

### Medical history

The patient had a history of grade 2 hypertension accompanied by deep venous thrombosis of the left lower extremity (intermuscular venous thrombosis of the left leg), bilateral carotid arteriosclerosis, and arteriosclerosis of both lower extremities.

### Physical and laboratory examinations

Physical examination revealed swelling of the upper left thigh, with no palpable mass or tenderness in the inguinal area. Local tenderness and knocking pain were present on the outside of the left hip joint. Further examination showed a positive Patrick's sign and a negative Thomas’ sign. The active and passive movements of the hip joint were limited, especially external rotation, and the patient displayed normal muscle tension and strength.

Routine laboratory tests, including blood, urine, and biochemical tests, yielded normal results. Regarding hematological and oncological indexes, the neuron-specific enolase level was slightly elevated at 15.40 μg/ml, the cancer antigen 72–4 level was 26.83 U/ml, and the ferritin level had increased to 230.90 ng/ml, which was a high value but still within the normal range. After excluding other lesions, these laboratory results were considered related to a malignant tumor's invasion of the bone marrow.

### Imaging examinations

**Figure 1:**
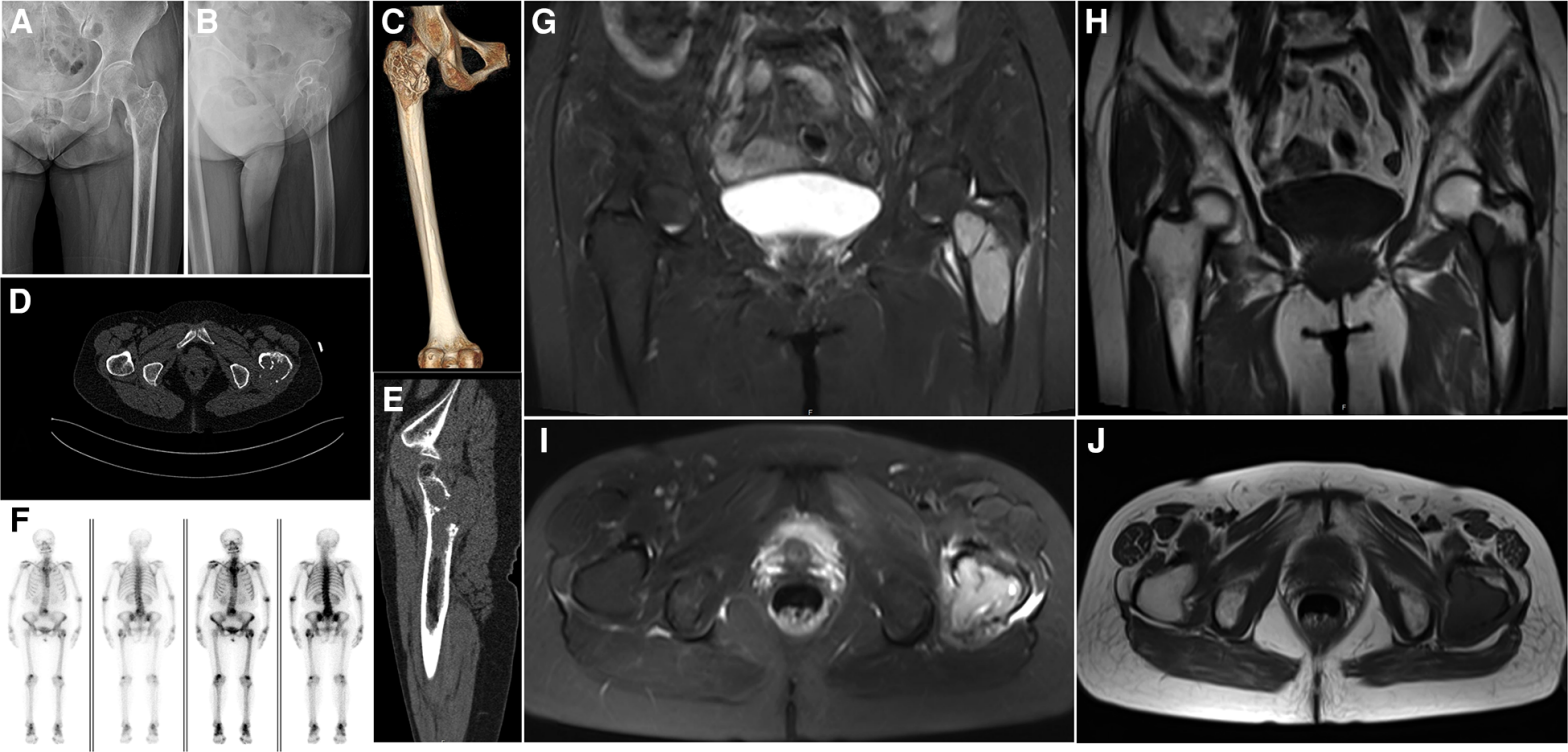
Preoperative imaging. (**A,B**) A radiograph of the left hip joint shows the extension of the tumor from the proximal femur to the subtrochanter of the femur; osteolytic lesions invading the bone cortex and proximal bone marrow cavity, as well as local osteosclerosis, can be observed. (**C**) Three-dimensional computed tomography (CT) reconstruction shows that the tumor is mainly located in the trochanteric area of the femur, with obvious bone erosion and insect erosion appearance. (**D,E**) A CT scan of the left hip shows that the tumor's lower and upper boundaries are clear and unclear, respectively, and osteolytic bone destruction can be observed. (**F**) A whole-body bone scan shows no other obvious positive areas. (**G**) A sagittal view of a T2-weighted image shows that the tumor is well-bounded and multilocular, with lytic destruction and the tumor extending outside the bone. (**H**) A sagittal view of a T1-weighted image illustrates that the tumor has a low signal shadow, similar to the muscle signal, and the area is larger than the plain film area. (**I**) A transverse view of a T2-weighted image shows uneven hypersignal shadows in the left trochanteric area with soft tissue infiltration on the posterolateral side of the trochanter. (**J**) A transverse view of a T1-weighted image shows that low signal shadows dominate the entire trochanteric area.

On November 26, 2017, a plain radiograph showed an osteolytic lesion with cystic destruction in the left femur's intertrochanteric region, and the lesion's boundary was clear without periosteal reaction (Figures 1A,B). Computed tomography (CT) showed that the tumor broke through the bone cortex and formed a significant soft tissue mass (Figures 1C–E). MRI revealed that the intertrochanteric medullary cavity of the left femur was occupied by short T1 and long T2 signals (Figures 1G–J). T1-weighted images showed that the tumor signal was similar to that of the muscle tissue, and the lesion included some parts of the femoral neck, trochanter, and subtrochanter. A whole-body bone scan showed that the intertrochanteric nuclides of the left femur were enriched and that bone salt metabolism had increased significantly; however, the bone salt metabolism of other parts of the body had not significantly increased (Figure 1F). Preoperatively, a CT scan of the head, neck, abdomen, and pelvis and a gynecological B-mode ultrasound revealed no metastasis in the gastrointestinal tract or uterus, and no signs of recent cerebral infarctions were present.

After obtaining informed consent from the patient and her family, a puncture biopsy of the left intertrochanteric bone tumor was performed on December 5, 2017. The pathological evaluation demonstrated a left femoral intertrochanteric leiomyosarcoma.

## Diagnostic assessment

### Final diagnosis

Histopathological analysis revealed a myogenic tumor showing characteristics consistent with those of LMSB. The results revealed a low-grade malignant tumor. Imaging showed an invasive growth breaking through the compartment, although there were no metastases. Therefore, the tumor was classified as G1T2M0 and staged as IB (extracompartmental low-grade malignant) according to the Enneking staging system. In this case, the final preoperative diagnosis was stage IB LMSB in the femur's trochanteric region. Extensive resection of the proximal femoral lesion was needed based on the above results.

### Treatment

**Figure 2:**
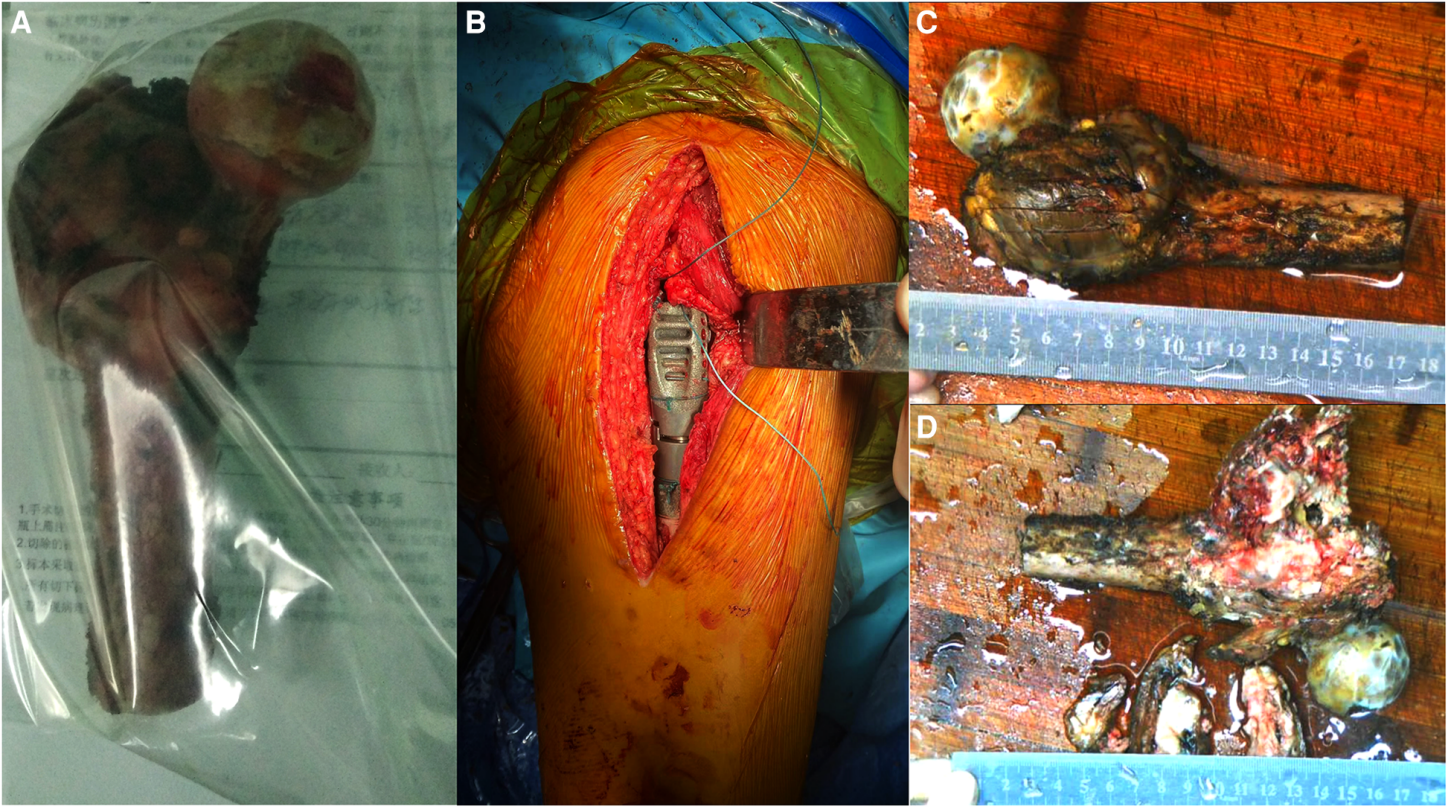
Intraoperative imaging and postoperative gross specimen. (**A,B**) The proximal femur is completely resected, and the tumor prosthesis is successfully implanted. (**C**) The gross specimen shows an uneven mass in the trochanter of the femur, with a size of approximately 6.0 cm × 6.0 cm × 4.0 cm. (**D**) The section of the specimen shows that the center of the mass is gray-white, with bleeding and necrotic areas invading the bone cortex.

We monitored the patient's general condition for 1 week preoperatively and performed detumescence, anticoagulation, and placement of a lower limb thrombus filter while monitoring heart, lung, and brain functions. We customized the prosthesis and performed extensive resection of the femoral intertrochanteric leiomyosarcoma and tumor prosthesis replacement under general anesthesia (Figure 2).

**Figure 3:**
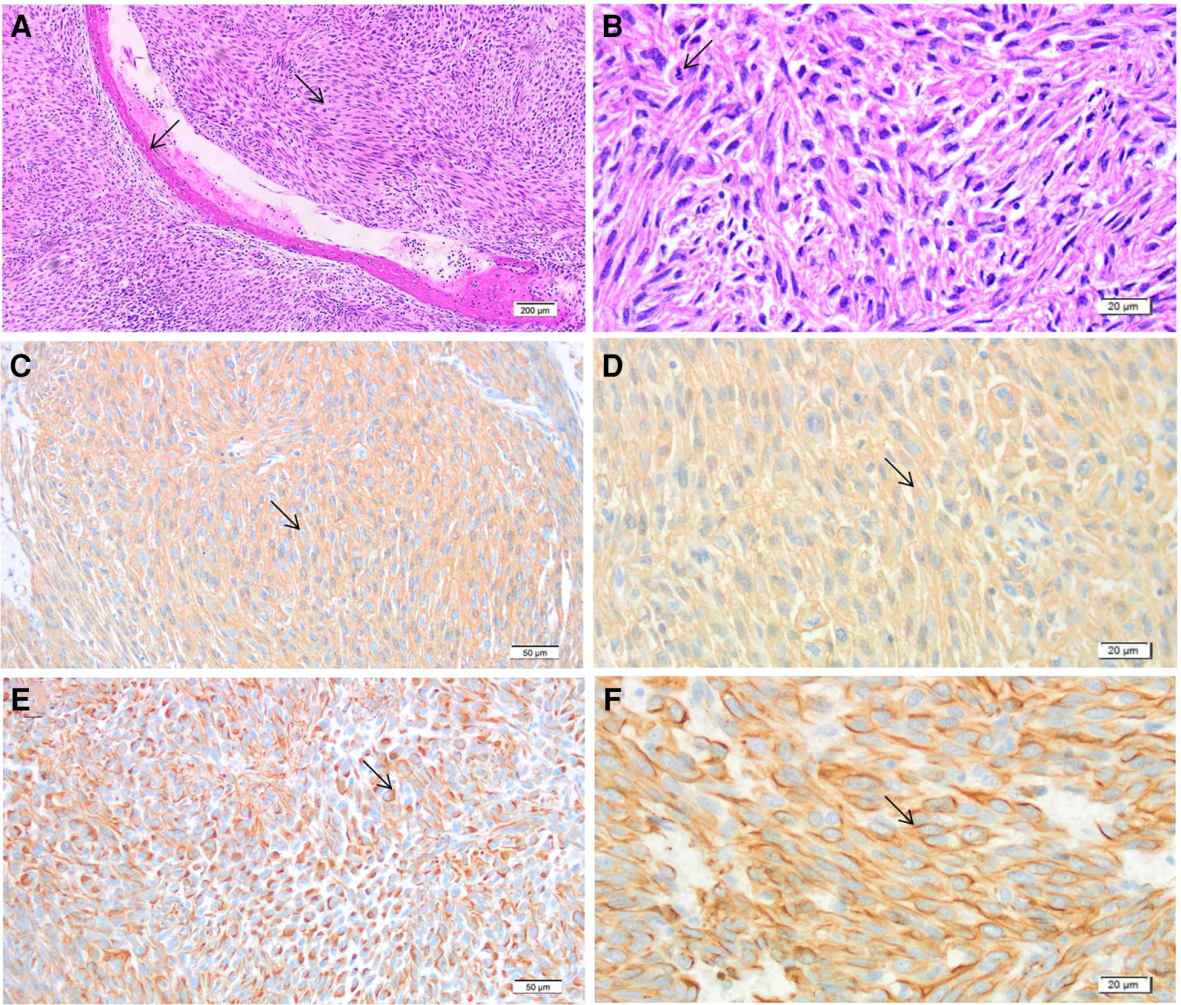
Results of postoperative pathological examination of the bone tumor. (**A**) Hematoxylin and eosin staining 40×: fusiform cell diffuse hyperplasia and tumor cells infiltrate adjacent bone trabeculae, as indicated by the arrow. Inflammation is observed around the bone trabeculae. (**B**) Hematoxylin and eosin staining 400×: fusiform cell diffuse hyperplasia with a myxohyaline matrix pervading the intercellular space, as indicated by the arrow. (**C**) Smooth muscle actin 200×: cytoplasmic staining is diffuse and strongly positive, as indicated by the arrow. (**D**) Smooth muscle actin 400×: cytoplasmic staining is diffuse and strongly positive, as indicated by the arrow. (**E**) Desmin 200×: some cytoplasmic staining is positive, as indicated by the arrow. (**F**) Desmin 400×: some cytoplasmic staining is positive, as indicated by the arrow.

A 15-cm incision was made on the posterior side of the left hip joint. The tumor was located in the greater trochanter of the femur, infiltrating the external circumflex muscle group. We resected the normal part of the external circumflex muscle group and incised the iliopsoas muscle downward at the lesser trochanter to expose the joint capsule. Next, we exposed the region below the lesser trochanter and peeled the gluteus minimus muscle upward to completely expose the incised articular capsule. Since the articular cartilage was in good condition and showed no infiltration, it was preserved. Based on the tumor involvement observed on the MRI scan, the femoral shaft was amputated 5 cm distal to the tumor, which was 16 cm from the apex of the greater trochanter. The attached muscle and proximal femur were completely removed. The cutting edge was subsequently found to be negative for tumor cells. Meanwhile, the size of the femoral head was 41 mm, and we selected an implant with a 40-mm bipolar femoral head. We sutured the joint capsule, reconstructed the muscles adjoining the proximal femur, and placed a drainage tube. The postoperative resected specimens were then sent for pathological examination (Figure 3).

### Outcome and follow-up

**Figure 4:**
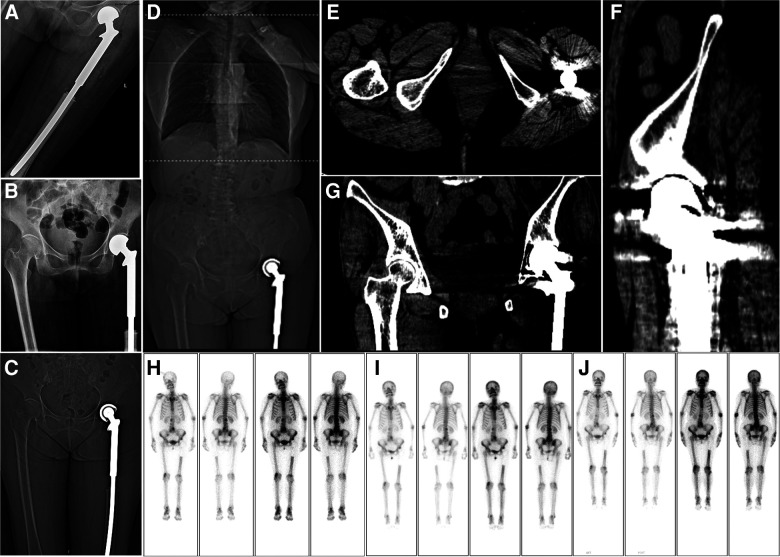
Postoperative imaging. (**A,B**) A radiograph of the left hip joint shows that the joint prosthesis is in place 1 week after the operation. (**C**) Coronal view of a computed tomography (CT) scan performed 1 week postoperatively. (**D–G**) Chest, abdominal, and pelvic CT examinations at 42 months postoperatively show no obvious metastasis in the lungs. Meanwhile, the joint prosthesis is in place, and there is no obvious recurrence in the local area. (**H–J**) After 13, 24, and 32 months postoperatively, whole-body bone scan findings show no obvious recurrence.

Two months postoperatively, the patient underwent five cycles of adjuvant chemotherapy. The chemotherapy regimen comprised cisplatin (30 mg/day) for 2 days, pirarubicin (50 mg/day) on the first day, and cisplatin (20 mg/day) on the third day. Routine clinical and radiological evaluations were performed every 3–4 months. Currently, 4 years postoperatively, the patient is in good physical condition and remains active, with no obvious pain in the hip joint or evident signs of tumor recurrence or metastasis (Figure 4). The patient's whole treatment and follow-up process is summarized in the timeline (Supplementary Figure S1).

## Discussion

LMSB is a rare and highly-invasive leiomyosarcoma that is difficult to diagnose and treat (7). The incidence of LMSB is slightly lower in men than in women (12), and the 5-year disease-specific survival rate of limb LMSB is 55%, while the median disease-specific survival after diagnosis is 61 months (95% confidence interval: 36–85 months) (15). Patients with metastasis at the initial diagnosis have worse prognoses (8). A previous study showed that the average age of patients with LMSB was 46 years (range: 9–88 years) (12). The optimal treatment for LMSB is unclear, and surgery, including tumor resection and limb salvage or hip amputation, is a widely-accepted treatment modality (15, 16). After successful limb salvage, the patient in this report was monitored for more than 50 months and showed no local recurrence or metastasis and satisfactory joint function. This case can serve as a reference for diagnosing and treating proximal femoral LMSB.

LMSB has a non-specific clinical presentation, as hip pain and pathological fractures shown by imaging are often the first symptoms (14). On plain radiographs, LMSB can appear as osteolytic, cystic, or mixed lesions and may include trabecular infiltration and periosteal reactions (17). Small bone infarctions are observed more clearly on CT scans than on radiographs and indicate small intraosseous vascular embolisms (18). The actual space occupation of LMSB lesions is larger than that observed *via* plain radiography, and the bone trabecula is disordered, which can be observed more clearly on MRI (19). LMSB tumors generally have low-signal shadows on T1-weighted images and high-signal shadows on T2-weighted images (19). LMSB occurs mostly in the long bones of the lower limbs and should be differentiated from other common bone tumors after the exclusion of metastases (7). LMSB commonly occurs in the same locations as osteosarcoma with more obvious bone destruction and periosteum reactions observed using radiography and CT, which are specific manifestations (20). However, the periosteal reactions of LMSB are not obvious. Myelomas may also present with lytic bone destruction, though osteoporosis can be observed on radiography and CT, and a myeloma includes multiple lesions with irregular shapes (21). LMSB, osteosarcoma, and myeloma appear as low-intensity lesions on T1-weighted images and high-intensity lesions T2-weighted images on MRI (7, 20, 21). Therefore, the specificity of the imaging results of LMSB is relatively low, and the diagnosis is dependent on pathological examination (8, 22).

The pathological examination of LMSB includes observing microscopic morphology and immunohistochemistry (19). Within LMSB lesions, long, narrow, spindle-shaped tumor cells form bundles within eosinophilic-rich cytoplasm and have cigar-shaped nuclei (23, 24). Immunohistochemistry is of unique value in diagnosing myogenic tumors and must be conducted to accurately diagnose LMSB (24).

Most LMSB lesions are positive for smooth muscle actin (SMA), approximately half are positive for intermediate filament protein (desmin), and some are positive for calmodulin (h-caldesmon). These important features of myogenic tumors have important auxiliary value for the diagnosis of LMSB (9, 14). LMSB is often misdiagnosed as metastatic bone leiomyosarcoma, undifferentiated pleomorphic sarcoma (UPS), fibroblastic osteosarcoma, and metastatic sarcomatoid carcinoma (7). Metastatic bone leiomyosarcoma is ruled out by the absence of primary lesions in the gastrointestinal tract or uterus *via* imaging (7, 15). The diagnosis of UPS, also known as high-grade malignant fibrous histiocytotumor, is typically exclusionary. UPS has no fixed morphologic arrangement and is positive for vimentin, CD68, and several other indicators as per immunohistochemistry (25). Therefore, the morphological characteristics and immunohistochemical results of UPS are not specific, though the malignancy of UPS is higher (26). Fibroblastic osteosarcoma tissues are arranged as fibrosarcomatoid structures and appear similar to UPS tissues under light microscopy, as a spindle cell sarcoma. However, focal malignant osteogenesis is observed in fibroblastic osteosarcoma lesions, and bone morphogenetic protein, osteocalcin, and osteoponectin positivity is observed on immunohistochemistry. No markers of myogenic tumors are observed in fibroblastic osteosarcoma lesions, distinguishing them from LMSB (7, 27). And SATB2 is a relatively specific marker of osteosarcoma which is also helpful in differentiating between LMSB and osteosarcoma (7, 27). Metastatic sarcomatoid carcinoma has a unique immunohistochemical feature as it expresses p63 and PAX8 and lacks the markers of myogenic tumors such as SMA and desmin. These characteristics distinguish metastatic sarcomatoid carcinoma from LMSB (7, 28). Previous studies have indicated the absence of malignant osteogenesis in LMSB, though calcification foci may be present, which can be used to distinguish LMSB from other conditions. In this patient, the sections of each part of the tumor were examined carefully, and no malignant osteogenesis was observed, which is consistent with the results of previous studies (7, 10). The lesion reported in this study presented as a spindle cell sarcoma under microscopy. The patients’ immunohistochemistry showed positive staining for SMA, desmin, vimentin, and p53. Metastatic tumors were excluded based on the patient's medical history and imaging results, and osteosarcoma was excluded based on the absence of malignant osteogenesis and positive immunohistochemical myogenic markers. The positive myogenic tumor markers also ruled out the diagnosis of metastatic sarcomatoid carcinoma. As the lesion appeared as a spindle cell sarcoma under light microscopy, the patient was diagnosed with LMSB.

Owing to the extremely low incidence of LMSB, effective treatments and prognostic factors remain unclear (7). Metastasis, delayed operations, and insufficient surgical margins are significantly associated with low overall survival rates (9). Early extensive surgical resection is necessary to overcome these challenges and has been proven to be an effective and radical cure for LMSB (15, 29). Active limb reconstruction can be performed following the extensive debridement of lesions (10). Mori et al. reported that negative surgical margins were associated with 2- and 5-year overall survival rates of 88.5% and 83.6%, respectively, highlighting the importance of surgical treatment and indicating that the clinical results of LMSB are affected by surgical margins (8). In another study, radiotherapy had no significant effects on the patients’ postoperative survival (30).

The choice of the surgical method is the primary focus when treating malignant bone tumors. Traditionally, amputation is combined with postoperative chemotherapy (31, 32). Amputation is considered when the tumor involves important vascular and nerve bundles and invades multiple compartments, when a severe pathological fracture is present, when the effect of simple chemotherapy is poor, or when the soft tissue is extensively involved (12). However, in recent years, limb salvage has been conducted more often (12). Currently, clinicians commonly use limb salvage surgical methods, including prosthesis reconstruction with tumor resection and prosthesis replacement and conventional internal fixation with a series of surgical procedures including lesion resection, bone cement filling, and implant fixation (12, 15, 32–38).

However, the revision of internal fixation surgery and prosthesis replacement for patients with malignant bone tumors may be necessary. The reoperation rate following custom-made tumor prosthesis replacement for metastatic bone tumors of the proximal femur may be lower than the reoperation rate following internal fixation. In a previous study, the quality of life of these patients who underwent prosthesis replacement was significantly improved, though the most common complication was prosthetic dislocation (11). The choice of the surgical method requires further studies assessing primary tumors of the proximal femur. The surgical margin is an important factor affecting the survival and prognosis in patients undergoing surgery without evidence of metastasis at the time of diagnosis (8). The purpose of surgery is to completely remove the gross and microscopic tumor tissue lesions, and limb salvage can be considered without reducing the survival rate (12). The incidences of re-fracture, bone nonunion, joint instability, and osteoarthritis are lower after prosthesis replacement than after allogenic bone and joint grafting (13).

The survival rate and prognosis of patients with LMSB are closely related to the diagnostic stage of the disease (12). The 5-year survival rate of patients with stage 1, 2A, and 2B tumors was as high as 60%, and the 10-year survival rate was 43% in a previous study. However, the prognosis of patients with stage 3 tumors was relatively poor, with a survival time of no more than 4 years and a median survival time of only 2 years (12). Therefore, timely diagnoses improve the patients’ prognoses (7, 8). The patient in this report had a good prognosis based on the tumor's low stage, a timely diagnosis, and active surgical resection of the tumor.

In conclusion, this report presents a rare case of grade IB LMSB in the trochanteric region of the femur in an older woman. Over the course of this patient's diagnosis and treatment, the pathological diagnosis was confirmed *via* puncture biopsy, and the tumor was graded and staged *via* imaging. The patient's joint function was successfully restored after an expanded tumor resection with negative margins and prosthesis replacement followed by postoperative chemotherapy. At the time of writing this report, the patient remained in a good physical condition with no apparent limitation of her hip functions and no obvious signs of local recurrence or distant metastasis of the tumor. This case report may serve as a reference for the clinical diagnosis and treatment of patients with Enneking grade IB LMSB of the proximal femur.

## Patient perspective

At first, the symptoms were too severe, and the pain in my left hip joint was quite unbearable. After the operation, my pain was relieved, and my joint activity improved significantly. However, there were some adverse reactions in the process of postoperative chemotherapy, which were fortunately resolved. I am very satisfied with the effect of the operation, and so far, there has been no recurrence or metastasis. My goal is to live for another 20 years.

## Data Availability

The original contributions presented in the study are included in the article/**Supplementary Material**, further inquiries can be directed to the corresponding author.

## References

[1] Evans DM Sanerkin NG. Primary leiomyosarcoma of bone. J Pathol Bacteriol. (1965) 90:348–50. 10.1002/path.1700900145 5843955

[2] Siegel RL Miller KD Jemal A. Cancer statistics, 2016. CA Cancer J Clin. (2016) 66:7–30. 10.3322/caac.21332 26742998

[3] Rigopoulou A Vlychou M Ostlere SJ Gibbons CLMH Athanasou NA. A primary leiomyosarcoma of bone containing pseudoepithelial plexiform elements. Skeletal Radiol. (2007) 36:791–6. 10.1007/s00256-007-0301-y 17483943

[4] Potsi M Stavrinou P Patsinakidis N Hatzibougias D Foroglou N Karayanopoulou G Primary osseous leiomyosarcoma of the spine: a rare entity—case report and review of the literature. J Neurol Surg A Cent Eur Neurosurg. (2012) 73:238–42. 10.1055/s-0032-1313588 20665430

[5] Adelani MA Schultenover SJ Holt GE Cates JMM. Primary leiomyosarcoma of extragnathic bone: clinicopathologic features and reevaluation of prognosis. Arch Pathol Lab Med. (2009) 133:1448–56. 10.5858/133.9.1448 19722754

[6] Recine F Bongiovanni A Casadei R Pieri F Riva N De Vita A Primary leiomyosarcoma of the bone: a case report and a review of the literature. Medicine. (2017) 96:e8545. 10.1097/MD.0000000000008545 29137065 PMC5690758

[7] Wang GY Lucas DR. Primary leiomyosarcoma of bone: review and update. Arch Pathol Lab Med. (2019) 143:1332–7. 10.5858/arpa.2019-0375-RA 31661313

[8] Mori T Nakayama R Endo M Hiraga H Tomita M Fukase N Forty-eight cases of leiomyosarcoma of bone in Japan: a multicenter study from the Japanese musculoskeletal oncology group. J Surg Oncol. (2016) 114:495–500. 10.1002/jso.24322 27302734

[9] Hanafy M Schwonzen M Kuhnen C Schley B Wilke A. Primary leiomyosarcoma of the distal fibula: a case report and review of the literature. Orthop Rev. (2018) 9:7236. 10.4081/or.2017.7236 PMC5850055 29564073

[10] Zumárraga JP Arouca MM Baptista AM Caiero MT Rubio DE de Camargo OP. Primary leiomyosarcoma of bone: clinicopathologic and prognostic factors analysis in a single institution. Acta Ortop Bras. (2019) 27:152–5. 10.1590/1413-785220192703215676 31452611 PMC6699396

[11] Di Martino A Martinelli N Loppini M Piccioli A Denaro V. Is endoprosthesis safer than internal fixation for metastatic disease of the proximal femur? A systematic review. Injury. (2017) 48:S48–54. 10.1016/S0020-1383(17)30658-7 29025610

[12] Brewer P Sumathi V Grimer RJ Carter SR Tillman RM Abudu A Primary leiomyosarcoma of bone: analysis of prognosis. Sarcoma. (2012) 2012:636849. 10.1155/2012/636849 22550421 PMC3329678

[13] Grinberg SZ Posta A Weber KL Wilson RJ. Limb salvage and reconstruction options in osteosarcoma. Adv Exp Med Biol. (2020) 1257:13–29. 10.1007/978-3-030-43032-0_2 32483727

[14] Rekhi B Kaur A Puri A Desai S Jambhekar NA. Primary leiomyosarcoma of bone—a clinicopathologic study of 8 uncommon cases with immunohistochemical analysis and clinical outcomes. Ann Diagn Pathol. (2011) 15:147–56. 10.1016/j.anndiagpath.2010.11.006 21393038

[15] Gusho CA Blank AT Gitelis S. Comparison of clinicopathological features and outcomes in patients with primary leiomyosarcoma of bone and soft tissue. J Surg Oncol. (2021) 123:1274–83. 10.1002/jso.26404 33524203

[16] Frings A Leithner A Liegl-Atzwanger B. Leiomyosarcoma of bone: a case report. Case Rep Med. (2011) 2011:980257. 10.1155/2011/980257 22174719 PMC3227273

[17] Bao RX. Radiologic-pathologic diagnosis of primary leiomyosarcoma of bone (a report of 7 cases). Zhonghua Fang She Xue Za Zhi. (1987) 21:82–5. PMID: 2961527 2961527

[18] Petra M Gibbons CLMH Athanasou NA. Leiomyosarcoma of bone arising in association with a bone infarct. Sarcoma. (2002) 6:47–50. 10.1080/13577140220127558 18521345 PMC2395491

[19] Goto T Ishida T Motoi N Yokokura S Kawano H Yamamoto A Primary leiomyosarcoma of the femur. J Orthop Sci. (2002) 7:267–73. 10.1007/s007760200045 11956991

[20] Nguyen JC Baghdadi S Pogoriler J Guariento A Rajapakse CS Arkader A. Pediatric osteosarcoma: correlation of imaging findings with histopathologic features, treatment, and outcome. Radiographics. (2022) 42:1196–213. 10.1148/rg.210171 35594197

[21] Weber M-A Baur-Melnyk A. Radiological diagnosis of multiple myeloma: role of imaging and the current S3 guideline. Radiologe. (2022) 62:35–43. 10.1007/s00117-021-00943-y 34919153

[22] Atalar H Gunay C Yildiz Y Saglik Y. Primary leiomyosarcoma of bone: a report on three patients. Clin Imaging. (2008) 32:321–5. 10.1016/j.clinimag.2007.10.022 18603190

[23] Sakumoto M Takahashi M Oyama R Takai Y Kito F Shiozawa K Establishment and proteomic characterization of NCC-LMS1-C1, a novel cell line of primary leiomyosarcoma of the bone. Jpn J Clin Oncol. (2017) 47:954–61. 10.1093/jjco/hyx096 28981730

[24] Yang Y Ma L Li L Liu H. Primary leiomyosarcoma of the spine: a case report and literature review. Medicine. (2017) 96:e6227. 10.1097/MD.0000000000006227 28248883 PMC5340456

[25] Matushansky I Charytonowicz E Mills J Siddiqi S Hricik T Cordon-Cardo C. MFH classification: differentiating undifferentiated pleomorphic sarcoma in the 21st century. Expert Rev Anticancer Ther. (2009) 9:113544. 10.1586/era.09.76 PMC3000413 19671033

[26] Goldblum JR. An approach to pleomorphic sarcomas: can we subclassify, and does it matter? Mod Pathol. (2014) 27:S39–46. 10.1038/modpathol.201.174 24384852

[27] Yoshikawa H Nakase T Myoui A Ueda T. Bone morphogenetic proteins in bone tumors. J Orthop Sci. (2004) 9:334–40. 10.1007/s00776-004-0764-9 15168194

[28] Yu W Yang L Wang J Gui L Li W Liu Z Case report: first case of consolidation immunotherapy after definitive chemoradiotherapy in mediastinal lymph node metastatic sarcomatoid carcinoma. Front Oncol. (2021) 11:788856. 10.3389/fonc.2021.788856 35083145 PMC8785342

[29] Gootee J Sioda N Aurit S Curtin C Silberstein P. Important prognostic factors in leiomyosarcoma survival: a national cancer database (NCDB) analysis. Clin Transl Oncol. (2020) 22:860–9. 10.1007/s12094-019-02196-7 31392646

[30] Antonescu CR Erlandson RA Huvos AG. Primary leiomyosarcoma of bone: a clinicopathologic, immunohistochemical, and ultrastructural study of 33 patients and a literature review. Am J Surg Pathol. (1997) 21:1281–94. 10.1097/00000478-199711000-00003 9351566

[31] Lapica H Ozery M Raju H Castro G de la Vega P R Barengo NC. The associations between racial disparities, health insurance, and the use of amputation as treatment for malignant primary bone neoplasms in the US: a retrospective analysis from 1998 to 2016. Int J Environ Res Public Health. (2022) 19:6289. 10.3390/ijerph19106289 35627824 PMC9140582

[32] Gaillard J Fouasson-Chailloux A Eveno D Bokobza G Da Costa M Heidar R Rotationplasty salvage procedure as an effective alternative to femoral amputation in an adult with a history of osteosarcoma: a case report and review. Front Surg. (2021) 8:820019. 10.3389/fsurg.2021.820019 35071319 PMC8776644

[33] Feng H Wang J Xu J Chen W Zhang Y. The surgical management and treatment of metastatic lesions in the proximal femur: a mini review. Medicine. (2016) 95:e3892. 10.1097/MD.0000000000003892 27428183 PMC4956777

[34] Liu T Guo X Zhang X Li Z Zhang Q. Reconstruction with pasteurized autograft for primary malignant bone tumor of distal tibia. Bull Cancer. (2012) 99:87–91. 10.1684/bdc.2012.1626 22863837

[35] Wang S Luo Y Zhang Y Wang Y Zheng C Tu C Case report: reconstruction of medialis malleolus (1/4 of the ankle joint) after resection of distal tibia tumor with an uncemented three-dimensional-printed prosthesis. Front Surg. (2022) 9:844334. 10.3389/fsurg.2022.844334 35402484 PMC8987288

[36] Kameda N Kagesawa M Hiruta N Akima M Ohki M Matsumoto T. Primary leiomyosarcoma of bone. A case report and review of the literature. Acta Pathol Jpn. (1987) 37:291–303. PMID: 3300160 3300160

[37] Bouaziz MC Chaabane S Mrad K Oueslati S Bellassoued A Ladeb MF Primary leiomyosarcoma of bone: report of 4 cases. J Comput Assist Tomogr. (2005) 29:254–9. 10.1097/01.rct.0000159581.54555.08 15772548

[38] Miura K Hatori M Hosaka M Kokubun S Watanabe M Ehara S. Primary leiomyosarcoma with the invasion into the intertrabecular space of bone: a case report and the review of the literatures. Clin Imaging. (2001) 25:209–14. 10.1016/s0899-7071(01)00249-2 11679231

