# Supplementary figures and images for: Case report and literature review: Primary leiomyosarcoma of the bone in the trochanteric region of the femur
Source: Front Surg. 2023 Jan 10;9:1045307. doi: 10.3389/fsurg.2022.1045307 (PMC9872517; doi:10.3389/fsurg.2022.1045307)

Supplementary Material


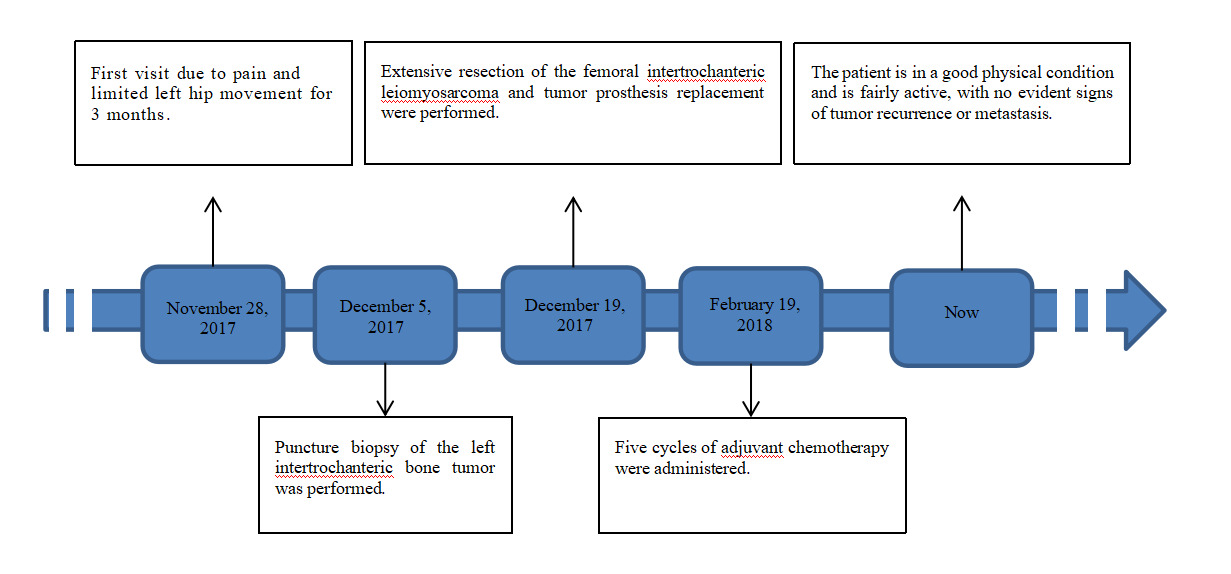


**Supplementary Figure 1.** Timeline of the patient’s treatment process.

Supplement: Supplementary file 1 [file Datasheet1.docx]
